# Novel Analytic Approaches to Investigate Minute-Level Actigraphy and Associations With Physical Function

**DOI:** 10.1093/geroni/igaa057.630

**Published:** 2020-12-16

**Authors:** Kaiyuan Hua, Sheng Luo, Katherine Hall, Miriam Morey, Harvey Cohen

**Affiliations:** 1 Duke University, Durham, North Carolina, United States; 2 Duke University, Veterans Health Administration, Durham, North Carolina, United States; 3 Veterans Health Administration, Durham, North Carolina, United States; 4 Duke University, School of Medicine, Durham, North Carolina, United States

## Abstract

Background. Functional decline in conjunction with low levels of physical activity has implications for health risks in older adults. Previous studies have examined the associations between accelerometry-derived activity and physical function, but most of these studies reduced these data into average means of total daily physical activity (e.g., daily step counts). A new method of analysis “functional data analysis” provides more in-depth capability using minute-level accelerometer data. Methods. A secondary analysis of community-dwelling adults ages 30 to 90+ residing in southwest region of North Carolina from the Physical Performance across the Lifespan (PALS) study. PALS assessments were completed in-person at baseline and one-week of accelerometry. Final analysis includes 669 observations at baseline with minute-level accelerometer data from 7:00 to 23:00, after removing non-wear time. A novel scalar-on-function regression analysis was used to explore the associations between baseline physical activity features (minute-by-minute vector magnitude generated from accelerometer) and baseline physical function (gait speed, single leg stance, chair stands, and 6-minute walk test) with control for baseline age, sex, race and body mass index. Results. The functional regressions were significant for specific times of day indicating increased physical activity associated with increased physical function around 8:00, 9:30 and 15:30-17:00 for rapid gait speed; 9:00-10:30 and 15:00-16:30 for normal gait speed; 9:00-10:30 for single leg stance; 9:30-11:30 and 15:00-18:00 for chair stands; 9:00-11:30 and 15:00-18:30 for 6-minute walk. Conclusion. This method of functional data analysis provides news insights into the relationship between minute-by-minute daily activity and health.

